# Conjugated linoleic acid ameliorates hepatic steatosis by modulating intestinal permeability and gut microbiota in ob/ob mice

**DOI:** 10.29219/fnr.v66.8226

**Published:** 2022-03-03

**Authors:** Shengli Gao, Yingying He, Liping Zhang, Lina Liu, Changfeng Qu, Zhou Zheng, Jinlai Miao

**Affiliations:** 1 Biomedical Center, Qingdao Medical College, Qingdao University, Qingdao, China; 2 Key Laboratory of Marine Eco-Environmental Science and Technology, First Institute of Oceanography, Ministry of Natural Resource, Qingdao, China; 3 Department of Special Medicine, School of Basic Medicine, Qingdao University; 4 Qingdao Key Laboratory of Marine Natural Products Research and Development, Qingdao, China; 5 Guangxi Academy of Sciences, Nanning, China

**Keywords:** conjugated linoleic acid, obesity, hepatic steatosis, intestinal permeability, gut microbiota

## Abstract

**Background:**

Conjugated linoleic acid (CLA) is an effective supplement for reducing fat mass, but its effect on hepatic steatosis remains controversial.

**Objective:**

This study aims to evaluate the effect of CLA on liver fat accumulation, inflammation, gut microbiome, and intestinal barrier integrity.

**Design:**

Wild-type (WT) mice and ob/ob (OB) mice were randomly divided into four groups according to the treatment with/without 1% CLA: WT, WT mice treated with CLA (WT-CLA), OB, and OB mice treated with CLA (OB-CLA). Lipid metabolism and hepatic fat accumulation were evaluated by changes in histological and biochemical parameters. Gene expressions related to liver inflammation and intestinal barrier integrity were examined. The effect of CLA on the gut microbiota population was investigated.

**Results:**

The body weight, fatty tissue mass, and serum lipid levels of the WT-CLA group and OB-CLA group were separately lower than those of the WT group and OB group, but the livers of the WT-CLA group had more fatty lipids, higher triglyceride properties, and saturated fatty acid (FA) composition than those of the WT group, which was contrary to the effect of CLA on OB mice. Real time quantitative PCR results showed that CLA increased hepatic inflammation and intestinal permeability in the WT mice, while it significantly decreased the mRNA expression of liver TNF-α, IFN-γ, and IL-1β and markedly ameliorated intestinal tight junction proteins in the OB mice. The gut microbiota testing indicated a higher abundance of beneficial bacteria (e.g., *Lachnoclostridium*, *Roseburia*, *Dubosiella*, *Oscillibacter*, and *Anaerostipes*) and a lower abundance of pro-inflammatory bacteria (e.g., *Tyzzerella* and *Alistipes*) in the OB-CLA group than those of the OB group. Correlation analysis suggested that gut microbiota correlated with liver inflammation, intestinal permeability, and hepatic FA composition.

**Conclusion:**

CLA potentially contributed to ameliorating hepatic steatosis in OB mice via modulating liver inflammation, intestinal permeability, and gut microbiota, which suggests CLA is more suitable for people with obesity or overweight.

## Popular scientific summary

- The effect of highly purified conjugated linoleic acid (CLA) on lipid metabolism was examined in wild-type and ob/ob mice.
- CLA supplementation potentially contributed to ameliorating the liver inflammatory state and fat accumulation in ob/ob mice.
- CLA administration partly reversed the increased intestinal permeability in ob/ob mice.
- CLA consumption led to changes in gut microbiome composition.
- These results could provide a new perspective on the role of dietary CLA in hepatic fat accumulation.

Conjugated linoleic acid (CLA) is a family of polyunsaturated fatty acids (FAs) characterized by two conjugated double bonds in different geometric (*cis*- or *trans*-) and positional sites. Of the 28 known isomers of CLA, *cis*-9, *trans*-11-CLA (c9t11-CLA), and *trans*-10, *cis*-12-CLA (t10c12-CLA) are the most abundant isomers in natural and synthetic CLAs (1). With an amazing effect on decreasing body weight through fat mass reduction, CLA has been widely used by obese people to lose weight (2). Nevertheless, CLA has been found to promote hepatic steatosis in rodent animals, which might be a potential threat to human health (3, 4). Some studies indicated that the potential mechanisms of hepatic steatosis induced by CLA were insulin resistance and consequent hyperinsulinemia, which was closely associated with decreased adipokine (leptin) (5–7). However, surprisingly, CLA alleviated hepatic steatosis in leptin-deficient ob/ob mice, which was reversed by the presence of leptin (8). Therefore, undefined mechanisms might be involved in hepatic steatosis induced by CLA.

The liver receives venous blood from the intestine, stomach, and pancreas via the portal vein, which contains nutrients and metabolites of host and microbiome origin (9). Through this route, enterically derived substances, such as lipopolysaccharide (LPS), may affect liver function and cause steatohepatitis by triggering Toll-like receptor 4 (TLR4)-dependent injury in the liver (10–12), which could be restrained by high-density lipoprotein (HDL) (13). CLA supplementation was found to modulate inflammatory cytokines, maintain the mucosal barrier, and revert microbiota changes in the intestines of mice with Dextran sulfate sodium (DSS)-induced colitis (14). Consequently, we speculate that CLA might play different roles in regulating the intestinal structure and gut microbiota in normal and ob/ob mice.

In this study, the effects of CLA on obesity and correlated metabolic disorders were investigated in ob/ob and normal mice. We studied changes in intestinal permeability and the microbiome induced by CLA. By comparing the differences between the ob/ob and normal mice, we aimed to delineate the possible mechanisms of hepatic steatosis induced by CLA.

## Materials and methods

### Reagents

The commercial CLA mixture was purchased from Qingdao Auhai Biotech Co., LTD (Shandong, China). SP-2560 Capillary GC Column (24056) was purchased from Merck KGaA (Darmstadt, Germany). Orlistat (ORL) was purchased from Cayman Chemical Co., Inc. (MI, USA). All serum biochemical test kits were purchased from the Nanjing Jiancheng Bioengineering Research Institute (Nanjing, China). All primers were obtained from Sangon Biotech (Shanghai, China). The PureLink RNA Mini Kit (12183018A), high-capacity cDNA Reverse Transcription Kit (4368814), DyNAmo ColorFlash SYBR Green qPCR Kit (F416S), Gene JETTM Gel Extraction Kit (K0692), and Ion Plus Fragment Library Kit (4471252) were purchased from Thermo Fisher Scientific (CA, USA). The QIAamp DNA Stool Mini Kit (Cat: 51504) was purchased from Qiagen (Hilden, Germany). The Phusion Flash High-Fidelity PCR Master Mix (F548S) was purchased from NEB (MA, USA). Male leptin-deficient ob/ob mice (T001461) and C57BL/6J wild-type mice (N000014) were purchased from Shanghai Biomodel Organism Science & Technology Development Company (Shanghai, China). The standard rodent diet (AIN93) was purchased from Qingdao Darenfucheng Animal Technology Co., Ltd. (Qingdao, China).

### CLA purification and gas chromatography analysis

The commercial CLA mixture was purified with urea-inclusion crystallization according to the Kim’s method (15). Briefly, the urea-saturated methanol solution was heated to 70°C for dissolving the CLA mixture and cooled down to room temperature in a cooling water bath. The obtained solution was then filtered (pore size: 0.45 μm) to remove urea-inclusion compounds, which contained most of the saturated FAs and monoenoic acids. The filtrate was added to the C urea-saturated methanol solution at 70°C. After the solution cooled to room temperature, CLA and urea formed CLA-urea-inclusion compounds, which were recovered when the solution mainly containing α-linolenic acid was filtered off. The compounds were solved with distilled water, and the highly purified CLA was mainly in the upper layer. Hexane extraction was used to recover CLA, which was prepared after being washed three times with distilled water. The purified CLA was analyzed by gas chromatography (GC) according to the protocol of the SP-2560 capillary GC column (16). The contents of different CLA isomers were calculated using the area normalization method.

### Animals and experimental design

Male leptin-deficient ob/ob mice (OB) and C57BL/6J wild-type mice (WT) aged 6 weeks were housed 4/cage at 23°C, with a 12 h light/12 h dark cycle and with a standard rodent diet and water *ad libitum*. After 1 week of adaptation, 20 WT mice and 20 OB mice were randomly divided into four groups as follows (*n* = 10): WT (olive oil, 1.5% body weight), WT-CLA (CLA, 1.5% body weight), OB (olive oil, 1.5% body weight), and OB-CLA (CLA, 1.5% body weight). Olive oil was used as a vehicle because of the similar uptake and metabolite distribution of CLAs and oleic acid (17). The CLA and the olive oil were administered by oral gavage at 9:00 am every day for 5 consecutive weeks. The body weight, 24-h food intake, and body length (from nose to anus) were recorded weekly. The Lee index of obesity was calculated as follows: the cube root of body weight (g) was divided by naso-anal length (cm) and multiplied by 1,000 (18). At the end of the experiment, each mouse was transferred to an empty cage to collect feces samples. The plasma was obtained from the retro-orbital plexus and centrifuged (4,000 rpm, 4°C, 40 min) to collect the serum samples. Fat tissue, liver, and colon were rapidly striped, weighed, and photographed. The feces, serum, and colon samples were stored at −80°C, and the adipose and liver tissues were fixed in 4% paraformaldehyde (PFA) for histological staining. All animal procedures complied with the China’s 109 Regulation on the Use and Care of Laboratory Animals and were approved by the Ethics Committee of Laboratory Animal Care of Qingdao University.

### Histological staining and cell size calculation

PFA-fixed fat and liver tissue sections were routinely stained with hematoxylin and eosin (H&E) (19). After dehydration and transparency, the samples were embedded in paraffin and cut into 7-μm-thick sections. Following deparaffinization and hydration, the sections were stained with H&E Y solution. After the tissue slides were prepared, the images were visualized using a BX63F microscope and captured with a DP80 digital camera (Olympus, Tokyo, Japan) (20).

Using ImageJ software (http://imagej.nih.gov/ij/), the cell size was calculated (19, 21). First, a known scale from the BX63F microscope was used to calibrate ImageJ. The photos were then opened and converted into gray-scale images in the software. The removal of background noise and unwanted particles significantly improved the clarity of these images. The membrane structure and blank space of the cells were identified with gray-scale threshold values. Lastly, the cell size was calculated using the free ‘Measure and Label Macro’ plugin for ImageJ (http://rsbweb.nih.gov/ij/plugins/measure-label.html) and displayed as the cell surface area (μm²). The shape parameter for cell selection was fixed between 0.35 and 1 (‘0’ refers to a straight line, while ‘1’ refers to a perfect circle). Cells touching the border of an image or shrunken cells were not counted. In every section, at least 200 adipocytes and 800 hepatocytes were used for the data analysis.

### Serum and liver lipid analysis

The levels of serum total cholesterol (TG) and triglyceride (TC) were measured using a TG or TC Assay Kit (glycerol 3 phosphate oxydase and phenol 4-aminoantipyrine peroxidase Method). The serum level of free fatty acid (FFA) was tested with an enzyme linked immunosorbent assay (ELISA) commercial kit. All operations were carried out according to the manufacturer’s instructions.

Total liver lipids were extracted using a mixture of methanol and chloroform (1:2, vol/vol) and resolved in a mixture of hexane: diethyl ether: acetic acid (70: 30: 0.2, by vol) for quantitative lipid class analyses in the thin-layer chromatography flame ionization detection system as previously described (22). Phospholipids (PL) were separated into classes by high-performance liquid chromatography (23). The FAs from the total PL, PL classes, cholesteryl esters (CE), and TG were esterified using sodium methylate and boron trifluoride, and quantitative data were analyzed using gas–liquid chromatography (24).

### Real-time quantitative PCR

Total RNA was extracted using a PureLink RNA Mini Kit, and cDNA synthesis was performed using a high-capacity cDNA Reverse Transcription Kit. Each reverse transcription PCR (RT-PCR) was carried out with a diluted cDNA template, forward and reverse primers (5 μm each), and a DyNAmo ColorFlash SYBR Green qPCR Kit (25). Real-time PCR was performed using the Applied Biosystems 7500 Fast Real-Time PCR System (Thermo Fisher Scientific, CA, USA). Primer sequences were as follows: TNF-α forward primer 5′-TAG CCA GGA GGG AGA ACA GA-3′, reverse primer 5′-TTT TCT GGA GGG AGA TGT GG- 3′; IFN-γ forward primer 5′-CCA ACG CAA AGC AAT ACA TGA-3′, reverse primer 5′-CGC TTC CCT GTT TTA GCT GC-3′; IL-1β forward primer 5′-TTG AAG AAG AGC CCA TCC TC-3′, reverse primer 5’-CAG CTC ATA TGG GTC CGA C-3’; IL-6 forward primer 5’-CCG GAG AGG AGA CTT CAC-3′, reverse primer 5’-TCC ACG ATT TCC CAG AGA-3’; occludin forward primer 5’-GGC AGC AGC TTG TTA AGC AG-3′, reverse primer 5’-ACT TGG CGC AGT GGT AAG CA-3’, zonula occludens-1 (ZO-1) forward primer 5′-GCC GCT AAG AGC ACA GCA A-3′, reverse primer 5′-TCC CCA CTC TGA AAA TGA GGA-3’; β-actin forward primer 5′-GAG ACC TTC AAC ACC CCA GC-3′, reverse primer 5′-ATG TCA CGC ACG ATT TCC C-3′. The cycle-threshold (Ct) value was automatically shown by the instrument’s software, and the relative mRNA expression level of each gene was calculated using the 2^−ΔΔCt^ method. The reference gene β-actin was used as a normalizer.

### Western Blot

The fresh intestinal tissue was homogenized in radioimmunoprecipitation lysis buffer (RIPA buffer) with the protease inhibitor phenylmethane sulfonyl fluoride (PMSF, ThermoFisher scientific, 36978, IL, USA). Total protein concentration was measured using the BCA Protein Assay Kit (ThermoFisher Scientific, 23225, IL, USA) following the manufacturer’s instructions; 20 μg total protein was loaded per lane and separated by 10% sodium dodecylsulfate (SDS)-polyacrylamide gel electrophoresis, and then was transferred onto 0.45 μm nitrocellulose (NC) membrane (0.45 μm, Bio-Rad Laboratories, 1620115, Hercules, CA) according to the standard protocols. After blocking for 1 h with 5% milk in TBST buffer, membranes were incubated with rabbit anti-OZ 1 antibody (1:500, AF5145) or rabbit anti-occludin antibody (1:500, 27260-1-AP) at 4°C overnight. After incubation with HRP Goat anti-Rabbit Ig (1:2000, Gab97200) for 2 h at room temperature, proteins were detected using enhanced chemiluminescence (ECL) reagent (Millipore, Billerica, MA).

### Lactulose/mannitol assay

Intestinal permeability was evaluated using lactulose/mannitol assay (L/M assay), which was performed as previously described (26). Briefly, fasted rats were gavaged with the lactulose (100 mg/mL) and mannitol (50 mg/mL) solution and put into metabolic cages. Rat urine was collected during the following 8 h. Then, the urine sample was centrifugated at 10,000 r/min for 10 min and added 5% acetic acid. After that, the sample was boiled, centrifuged, and filtered to remove impurities. At last, the purified specimen was analyzed with high-performance liquid chromatography to calculate the ratio of lactulose and mannitol (L/M ratio). The change of intestinal permeability was indicated by the urine content of L/M leaked from the intestine.

### Analysis of gut microbiota

The total genomic DNA of the fecal samples was extracted using a QIAamp DNA Stool Mini Kit; 16S rRNA gene V3-V4 regions were amplified with specific primers 343F (5′-TACGGRAGGCAGCAG-3′) and 798R (5′-AGGGTATCTAATCCT-3′) following a previously described method (27). All PCRs were performed using a Phusion Flash High-Fidelity PCR Master Mix, and mixed PCR products in equal quantities were purified using a Gene JETTM Gel Extraction Kit. Sequencing libraries were acquired with an Ion Plus Fragment Library Kit. Lastly, the library was sequenced on an Ion S5 TM XL platform, and 400 bp/600 bp single-end reads were generated (Novogene Co., Ltd. Beijing, China). After sequencing, the 16S rRNA sequence data were analyzed using bioinformatics methods, as previously described (28). UPARSE software (Uparse v7.0.1001, http://drive5.com/uparse/) assigned the sequences with ≥97% similarity to the same operational taxonomic units (OTUs). The representative sequence of every OTU was annotated taxonomic information according to the Silva Database (Version 132, https://www.arbsilva.de/). The principal coordinate analysis (PCoA) and microbial diversity were performed via Novomagic (https://magic.novogene.com).

### Statistical analysis

The results were expressed as the mean ± standard deviation (SD). Body weight and food intake were analyzed using repeated-measures mixed models, assuming unstructured covariance. For other data, Student’s *t*-test was used to compare differences between two groups, and a one-way analysis of variance (ANOVA), followed by a *post hoc* Tukey’s test, was used to determine differences between more than two groups. The Spearman’s correlation coefficient was calculated with GraphPad Prism 8 (La Jolla, CA, USA), and the clustering results were visualized with heatmaps constructed and analyzed using Cluster 3.0 and Java Treeview software 1.6. Values of *P* < 0.05 were considered statistically significant.

## Results

### Effects of CLA on fat mass and liver

**Fig. 1:**
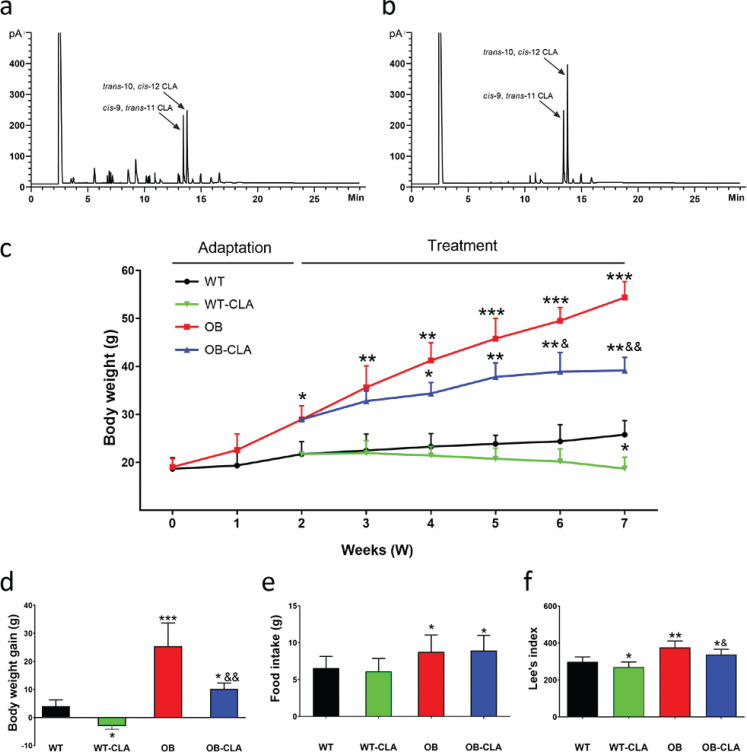
Meteorological chromatograms and weight loss effects of CLA. The meteorological chromatograms of crude CLA (a) and highly pure CLA (b) were showed. The changes in body weight (c), body weight gain (d), food intake (e), and Lee’s index (f) of mice during the 7-week CLA supplementation are displayed. The data are expressed as means ± SD. Comparison with the WT group, *P < 0.05, **P < 0.01, ***P < 0.001; comparison with the OB group, &*P* < 0.05, &&*P* < 0.01, &&&*P* < 0.001; (*n* = 8).

Currently, the commercially available CLA product is commonly synthesized from linoleic acid by alkaline isomerization (29). In this study, the crude CLA mixture was purified using the method of urea-inclusion crystallization (15). According to the GC analysis results in Fig. 1a and b, the purity of CLA increased from 70.21 to 94.68%. The highly purified CLA included 35.84% c-9, t-11 CLA, and 56.25% t-10, c-12 CLA, which suggested that the function of CLA was more similar to that of t-10, c-12 CLA.

To study the effect of highly pure CLA on the food intake and body weight of OB mice, we recorded the food consumed in 24 h and body weight once per week. At the end of the 2nd week, the average body weight of the OB group was significantly higher than that of the WT group (Fig. 1c). This showed that ob/ob mouse was an excellent genetically obese model to assess the effects of CLA on congenital obesity. After a 5-week treatment, body weight was noticeably decreased in both WT-CLA (*P* < 0.05) and OB-CLA (*P* < 0.01) groups (Fig. 1c), and the results of body weight gain showed the same trend (Fig. 1d). There was no significant difference in food intake between the WT and WT-CLA or OB and OB-CLA groups (Fig. 1e), which suggested that the effect of CLA on body weight might be due to increased fat metabolism, not appetite reduction. Lee’s indexes for assessing obesity in the WT-CLA and OB-CLA groups were significantly decreased compared with the WT (*P* < 0.05) and OB (*P* < 0.05) groups, respectively (Fig. 1f). These results demonstrated that CLA prevented body weight gain in both wild-type and ob/ob mice and was more effective on weight loss in the obese mice.

**Fig. 2:**
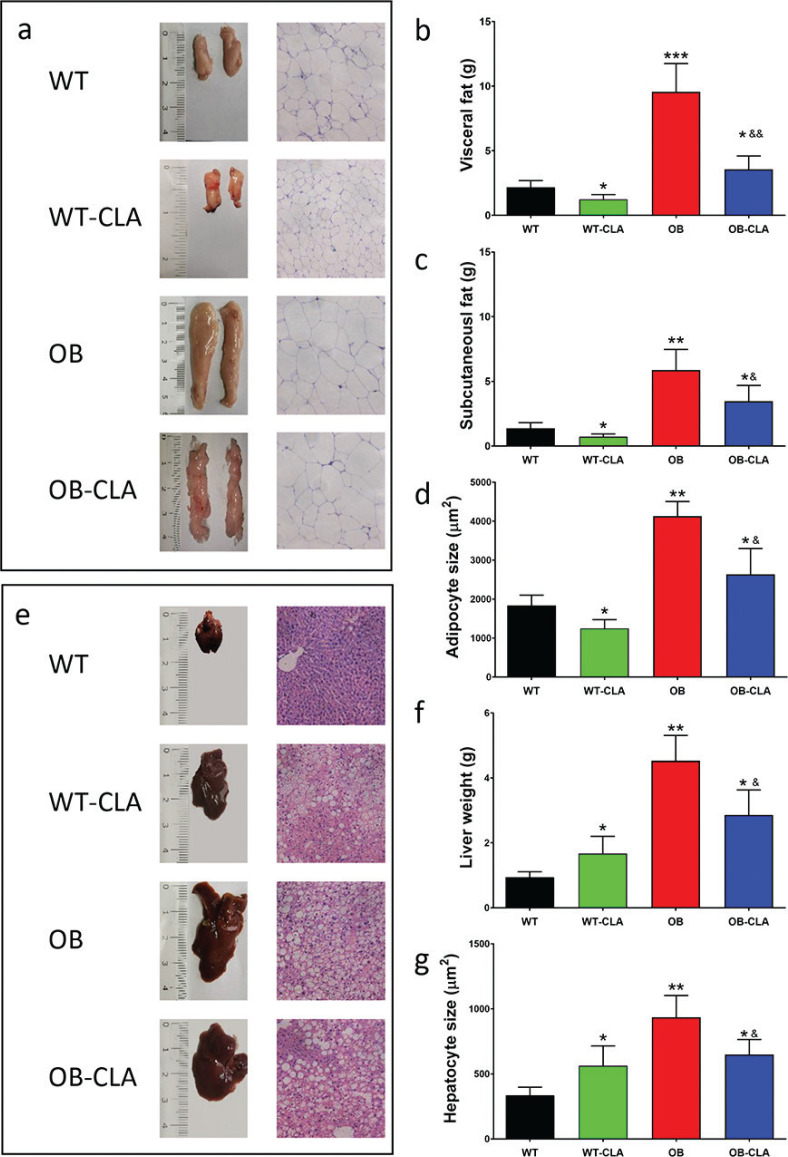
Effects of CLA on fat tissue and liver. CLA supplementation significantly reduced the volume of fat mass (a), the weight of visceral fat (b), and subcutaneous fat (c) in the WT and OB mice. The H&E staining of fat mass (a) and adipocyte size measurement (d) showed the effect of CLA on the adipocytes. The effects of CLA on the livers of WT and OB mice were shown: (e) hepatic morphology and H&E staining, liver weight (f), and hepatocyte size (g). The data are expressed as means ± SD. Comparison with the WT group, *P < 0.05, **P < 0.01, ***P < 0.001; comparison with the OB group, &P < 0.05, &&P < 0.01; (n = 8).

CLA supplementation induced a decrease in fat tissue mass in both the WT and OB mice (Fig. 2a). The weight of visceral and subcutaneous fat also showed dramatic effects of CLA (Fig. 2b and c). H&E staining of fat tissue further confirmed the anti-obesity effect of CLA, with smaller adipocytes in the WT-CLA and OB-CLA groups than in the WT and OB groups separately (Fig. 2a and d).

We investigated the effect of CLA on hepatic steatosis, observing that OB mice had a larger liver than the WT mice. CLA treatment enlarged the liver of the WT mice but reduced the size of the liver in the OB mice (Fig. 2e). The liver weight was significantly increased by CLA in the WT-CLA group compared with the WT group (*P* < 0.05) and was remarkably reduced in the OB-CLA mice (*P* < 0.01, Fig. 2f), which was consistent with the different hepatocyte sizes of the four groups, as shown by the H&E staining (Fig. 2e and g). Therefore, CLA significantly decreased fat mass in both WT and OB mice but induced different effects of hepatic lipid accumulation in the WT and OB mice.

### Effects of CLA on serum and liver lipids in OB mice

**Fig. 3:**
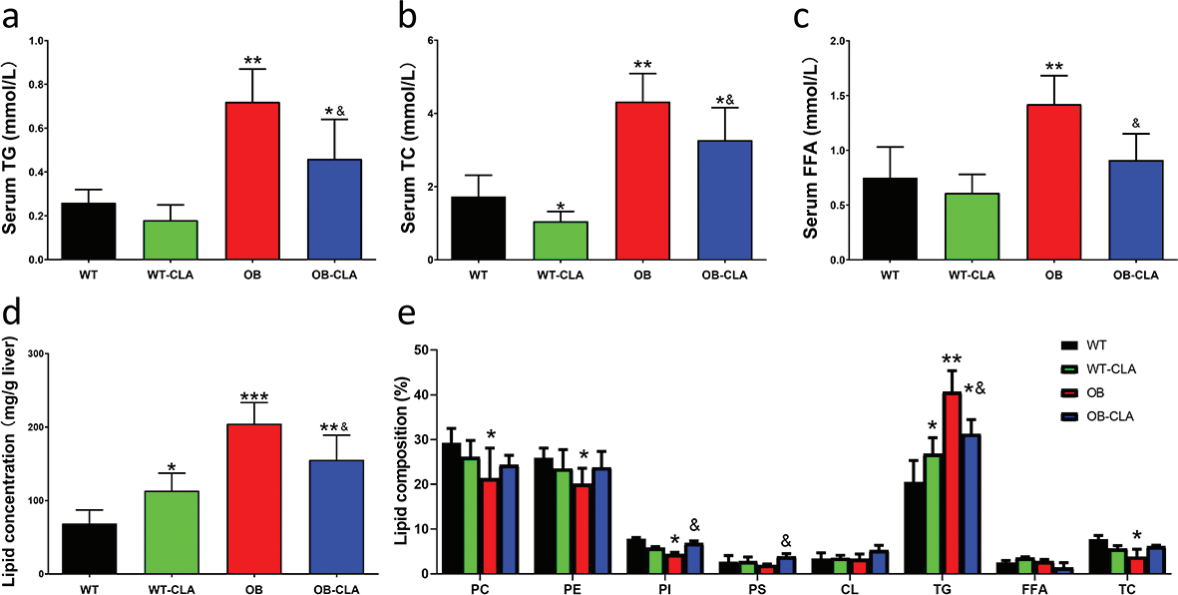
Effects of CLA on serum lipid levels and liver lipid composition. Serum lipids were measured, including TG (a), TC (b), and FFA (c), and were decreased by CLA in OB mice. CLA increased the lipid concentration of the liver in the WT mice, but decreased the lipid concentration in the OB mice (d). CLA modified the lipid composition of the liver in the WT and OB mice (e). The data are expressed as mean ± SD. Comparison with the WT group, *P < 0.05, **P < 0.01, ***P < 0.001. Comparison with the OB group, &*P* < 0.05; (*n* = 8).

The serum lipid concentrations of the OB group were significantly higher than those of the WT group (Fig. 3a–c), which suggested that the OB mice had hyperlipidemia. CLA treatment notably decreased the serum levels of TG, TC, and FFA of the OB-CLA group compared with those of the OB group (all *P* < 0.05, Fig. 3a–c). For the WT mice, CLA showed few effects on serum lipids compared with the OB mice (Fig. 3 a–c).

Liver lipids were extracted and analyzed, revealing that the amount of liver lipids in the WT-CLA group was significantly higher than that of the WT group (*P* < 0.05), while CLA markedly decreased the lipid content of the OB-CLA group compared with the OB group (*P* < 0.01, Fig. 3d). The lipid composition of the livers is shown in Fig. 3e. CLA significantly increased the triacylglycerol content of the liver (26.84 ± 3.59%) in the WT-CLA group compared with the WT group (20.54 ± 4.78%, *P* < 0.05), but the triacylglycerol was markedly decreased in the OB-CLA group (31.28 ± 3.19%), which was mainly balanced by the phospholipid content. The proportion of total cholesterol and free FAs in the liver was not affected by CLA in either the WT or OB mice. Table 1 presents the FA composition of the liver lipids. The proportions of long-chain saturated fatty acids (LCSFAs, 14:0, 16:0, and 18:0) were higher in the WT-CLA group compared with the WT group (*P*s < 0.05), but were decreased in OB-CLA mice (*P*s < 0.05). The proportion of unsaturated FAs (e.g., 20:4;5,8,11,14 and 18:2;9,12) was significantly reduced by CLA in the WT mice (*P* < 0.05), while it was dramatically increased in the OB mice (*P* < 0.05). CLA administration significantly elevated the proportion of 18:2 (9,11 and 10,12) in the liver FA of both WT and OB mice (*P* < 0.0001). These data suggest that CLA modified the composition of liver lipids and FAs in WT and OB mice.

**Table 1:** Long-chain fatty acid composition (%) of liver lipids in the mice fed CLA

Fatty acid	WT	WT-CLA	OB	OB-CLA
14:0	0.21 ± 0.08	0.48 ± 0.25*	0.43 ± 0.23	0.27 ± 0.15^&^
16:0	22.01 ± 3.38	27.54 ± 4.88*	29.01 ± 6.1	20.46 ± 3.39^&^
16:1	2.39 ± 0.5	3.14 ± 0.89	4.14 ± 0.51	4.23 ± 0.61
18:0	4.92 ± 2.32	6.63 ± 2.68*	6.16 ± 3.23	3.85 ± 0.39^&^
18:1	13.88 ± 3.61	14.18 ± 3.24	26.26 ± 4.37	18.59 ± 3.65^&^
18:2;9,12	18.57 ± 1.23	14.56 ± 2.27*	11.64 ± 1.96	15.84 ± 2.97^&^
18:2 (9,11-CLA)	0.09 ± 0.04	1.05 ± 0.19***	0.02 ± 0.02	1.82 ± 0.29^&&&^
18:2 (10,12-CLA)	0.02 ± 0.01	1.44 ± 0.24***	0.04 ± 0.03	2.38 ± 0.05^&&&^
20:3	2.38 ± 0.25	2.41 ± 0.17	2.52 ± 0.18	2.15 ± 0.18
20:4;5,8,11,14	12.44 ± 5.18	5.01 ± 3.25*	7.64 ± 3.79	13.74 ± 3.02^&^
22:6	13.08 ± 3.18	11.25 ± 2.37	10.37 ± 4.8	12.46 ± 2.38

Comparison with the WT group,

* *P* < 0.05,

*** *P* < 0.001.

Comparison with the OB group,

& *P* < 0.05,

&&& *P* < 0.001.

### Effects of CLA on hepatic inflammatory cytokines and intestinal permeability

**Fig. 4:**
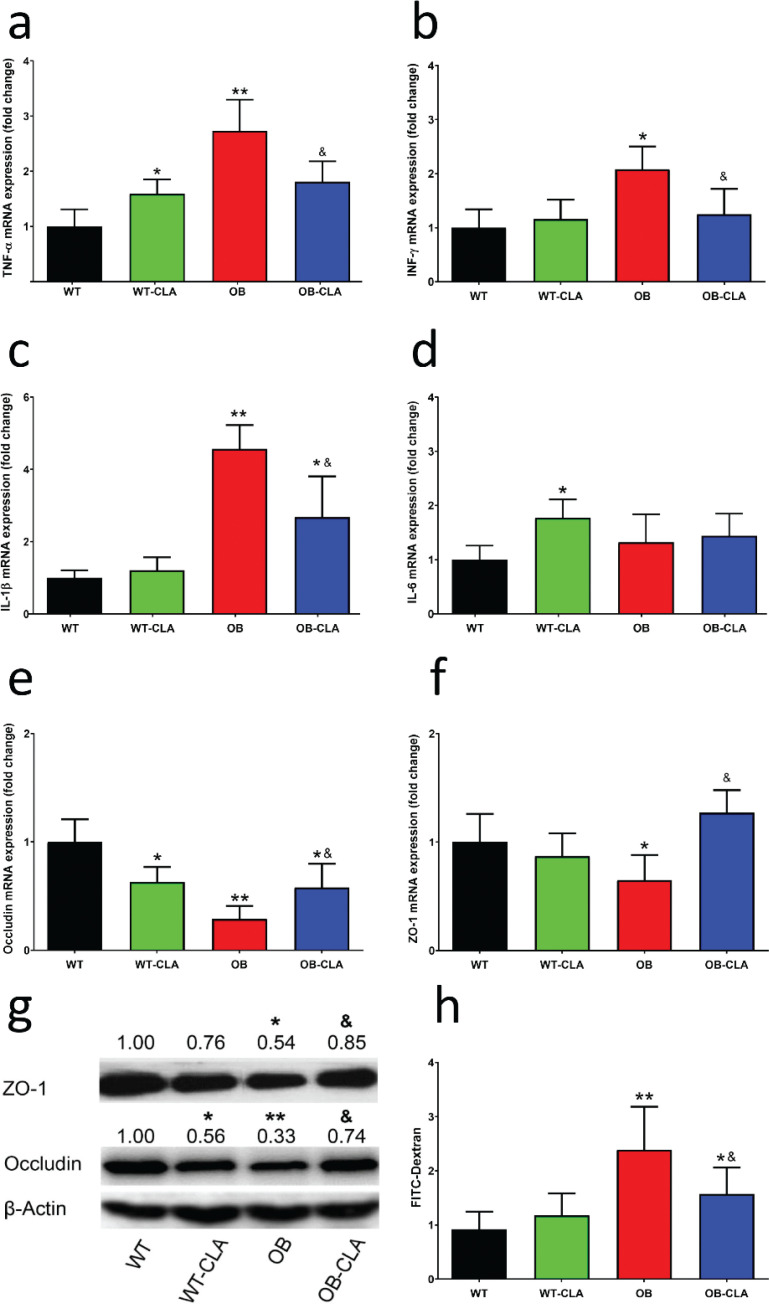
Effects of CLA on the mRNA expression of inflammatory cytokines and intestinal permeability. CLA treatment influenced the mRNA-expressing levels of TNF-α (a), IFN-γ (b), IL-1β (c), and IL-6 (d) in the liver. The mRNA expression levels of the intestinal tight junction proteins occludin (e) and ZO-1 (f) in colon tissue were measured using RT-qPCR. The protein expression levels of occludin and ZO-1 in colon tissue were detected with western blot (g). Lactulose/mannitol assay was used to check the intestinal permeability (h). The data are expressed as mean ± SD. Comparison with the WT group, *P < 0.05, **P < 0.01; comparison with the OB group, &*P* < 0.05; (*n* = 8).

Inflammation is an important risk factor of hepatic steatosis, so the mRNA expression of several important inflammatory cytokines in the liver was examined. In the WT mice, CLA enhanced the gene expression of TNF-α and IL-6 in the liver (*P* < 0.05, Fig. 4a and d). The increased gene expression levels of TNF-α, IFN-γ, and IL-1β in the OB mice were significantly inhibited in the OB-CLA group (*P* < 0.05, Fig. 4a–c). The liver mainly receives venous blood from the intestine via the portal vein, which contains nutrients and other substances that are absorbed in the intestines. Thus, hepatic inflammation might be related to intestinal permeability.

Intestinal permeability is closely associated with changes in tight-junction structural proteins (30), involving occludin and ZO-1. RT-PCR investigation revealed that the mRNA expressions of occludin and ZO-1 in the OB group were significantly lower than that of the WT group (*P* < 0.01 and *P* < 0.05, Fig. 4e and f). CLA treatment decreased mRNA expressions of occludin in the proximal colon in the WT mice (*P* < 0.05, Fig. 4e), while it increased the lower occludin and ZO-1 mRNA level in the OB mice (*P* < 0.05, Fig. 4e and f). The protein expression of occludin and ZO-1 in the colon was further detected by western blot. The results showed that the OB mice had lower occludin and OZ-1 expression than that of the WT mice (*P* < 0.01 and *P* < 0.05, Fig. 4g), which was markedly restored by CLA treatment (*P* < 0.05, Fig. 4g). While for the WT mice, CLA depressed the expression of occludin protein in the WT-CLA group (*P* < 0.05 vs. WT group, Fig. 4g).

The lactulose/mannitol assay was used to assess intestinal permeability. As shown in Fig. 4h, the L/M ratio of OB group was significantly higher than that of the CON group (*P* < 0.01), indicating that the intestinal permeability was greatly increased in obesity mice. CLA treatment obviously decreased the L/M ratio of OB-CLA group (*P* < 0.05 vs. OB group), but had little effect on the mice of CON-CLA group. It suggested that CLA may be able to reduce the damage of intestinal epithelial tight junctions induced by obesity, possibly changing the contents of the hepatic portal vein.

### Effects of CLA on gut microbiota

**Fig. 5:**
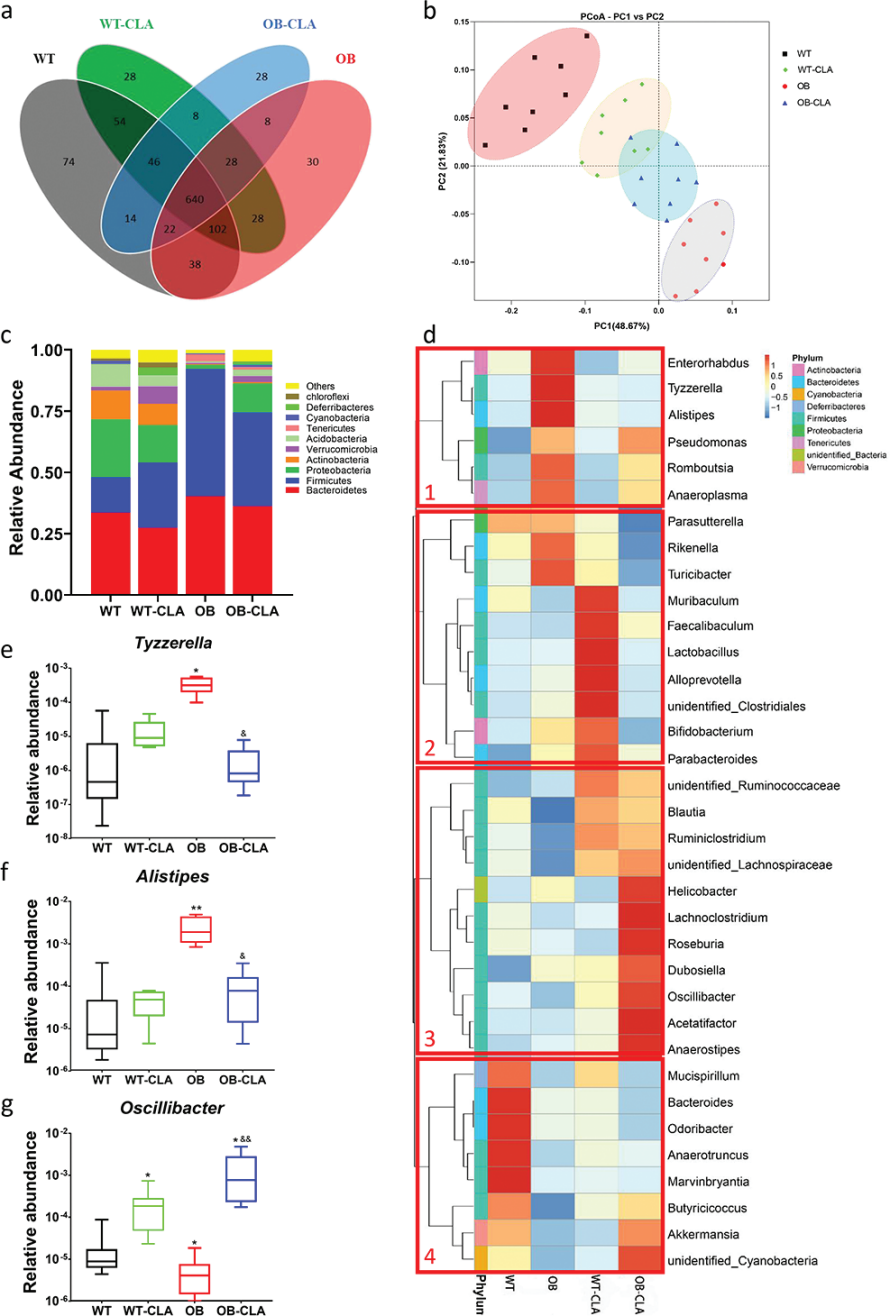
Effects of CLA on the gut microbial community. Collected fecal samples were analyzed based on the 16S rRNA sequence V3-V4 regions, and the results were shown as follows: (a) OTU cluster analysis of the gut microbiota, (b) the modified gut microbial community after CLA treatment, (c) the 10 dominant microbial communities at the phylum level in different groups, and (d) the microbial community bar plot at the genus level. The relative abundances of three typical bacteria (*Tyzzerella*, *Alistipes*, and *Oscillibacter*) are displayed (e).

Gut microbiota play important roles in lipid metabolism and absorption. Here, we collected feces samples to analyze the composition of gut microbiota with regard to CLA modulation. The OTUs were clustered with a standard of 97% similarity according to 16S rRNA sequences, identifying 574 OTUs. To describe sample similarity and overlap, the number of common and unique OTUs is shown in a Venn diagram (Fig. 5a). The unique and total numbers of OTUs in the WT, WT-CLA, OB, and OB-CLA groups were 74/990, 28/794, 28/934, and 30/896, respectively. Thus, the ratios of unique to total OTUs of each group were 7.47, 3.53, 3.00, and 3.35%, respectively. The PCoA of the weighted UniFrac distance displayed four completely separate clusters (Fig. 5b). The results revealed that the gut microbiota of the OB mice were dramatically different from that of the WT mice (PC1, 48.67%). CLA treatment partly reversed the shift in gut microbiota in the OB mice (PC2, 21.83%, Fig. 5b). Based on the clustering results, CLA supplementation altered the community of gut microbiota in the WT and OB mice and improved the diversity of gut microbiota to a normal level.

The effects of CLA on gut microbiota composition and the relative abundances of the bacterial species, especially dominant ones, at different levels of classification were assessed. At the phylum level, the three major phyla of gut microbiota in the WT group were *Bacteroidetes*, *Firmicutes*, and *Proteobacteria*. There were significant increases in the relative abundance of *Firmicutes* (51.86%) in the OB group compared with the WT group (14.42%, Fig. 5c). The ratio of *Firmicutes*/*Bacteroidetes* (F/B) of the OB group was notably higher than that of the WT group (1.2875 vs. 0.4296, *P* < 0.01), which was decreased by CLA treatment in the OB-CLA group (1.0580, Fig. 5c). Definitely, the relative abundance of *Proteobacteria* was recovered in the OB-CLA group (11.78%), which was remarkably decreased in the OB group (1.68%).

The effect of CLA supplementation on the relative abundance of gut microbiota was also observed at the genus level. The top 35 most-abundant genera were presented in a heat map and were classified into four different clusters according to the change in different components (Fig. 5d). In cluster 1, the OB group showed higher levels of some pro-inflammatory bacteria (e.g., *Enterorhabdus*, *Tyzzerella*, *Alistipes*, and *Rikenella*), which might be high-risk pathogens that increase intestinal permeability (31–33). CLA supplementation significantly decreased the abundance of these bacteria in the OB-CLA group, suggesting that CLA probably reduced the reproduction of these pro-inflammatory bacteria. In cluster 2, the WT-CLA group had higher proportions of *Muribaculum*, *Faecalibaculum*, and *Lactobacillus* than the WT group. In cluster 3, CLA treatment significantly increased well-known beneficial bacteria, such as *Lachnoclostridium*, *Roseburia*, *Dubosiella*, *Oscillibacter*, and *Anaerostipes*, in the OB-CLA group. Cluster 4 showed that the proportions of *Bacteroides, Odoribacter, Anaerotruncus*, and *Marvinbryantia* in the WT-CLA group were significantly lower than that in the WT group, while CLA supplementation was not so effective in the OB mice.

For a clearer change in genus, the abundances of three representative bacteria were further analyzed. The typical pro-inflammatory bacteria,*Tyzzerella* and *Alistipes*, were more abundant in the OB group compared with the WT group, which was reversed in the OB-CLA group (Fig. 5e and f). Another beneficial bacterium, *Anaerostipes*, was obviously increased after CLA supplementation in the WT and OB mice (Fig. 5g).

### Correlation between gut microbiota and hepatic steatosis

**Fig. 6:**
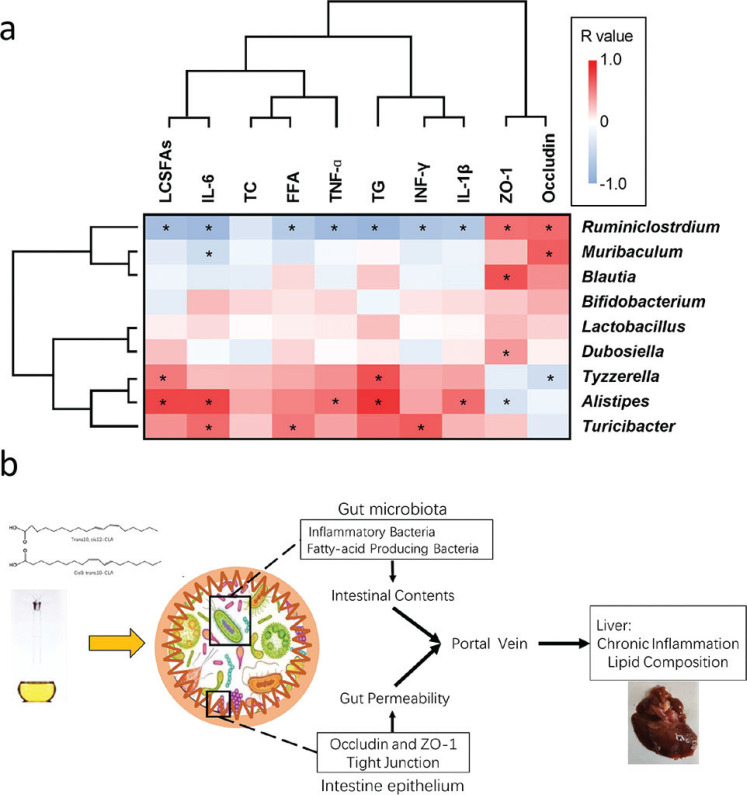
Correlation analysis between physiological data and gut microbial composition at the genus level. (a) The association between physiological data and gut microbial composition was displayed in a heatmap. (b) The scheme describes how CLA affects hepatic steatosis via a multifactor pattern.

Spearman’s correlation coefficient was used to analyze the connection between intestinal bacteria and hepatic steatosis. The correlation heat map (Fig. 6a) showed that the bacteria *Tyzzerella*, *Alistipes*, and *Turicibacter* had a widely positive correlation with liver lipids (TG and LCSFAs) and inflammation (gene expression of TNF-α, IFN-γ, IL-1β, and IL-6), but displayed a negative relationship with the intestinal tight junction (gene expression of occludin and ZO-1). *Ruminiclostridium, Muribaculum*, and *Blautia* showed a strongly positive correlation with intestinal tight junction, and *Ruminiclostridium* was negatively correlated with liver lipids and inflammation. These results indicate that gut microbiota were closely associated with liver inflammation, lipid levels, and intestinal permeability, which might contribute to hepatic steatosis in the model mice.

## Discussion

In this study, highly purified CLA was prepared using urea-inclusion crystallization. The normal lipid accumulation in the liver cells of normal rats was increased by CLA supplementation in the WT-CLA group, while the excess hepatic lipid accumulation in the ob/ob mice was significantly decreased in the OB-CLA group. Although the size of hepatocytes in the OB-CLA group was still larger than that of the WT-CLA group, CLA potentially contributed to ameliorating the hepatic steatosis in obese mice. Liver lipid analysis showed that there were different lipid classes and FFA compositions in the WT-CLA and OB-CLA groups. CLA promoted hepatic inflammatory and intestinal permeability in the WT mice, but it decreased the mRNA expression of liver TNF-α, IFN-γ, and IL-1β and improved the lower intestinal tight junction proteins in the OB mice. The gut microbiota test indicated a higher abundance of beneficial bacteria (e.g., *Lachnoclostridium*, *Roseburia*, *Dubosiella*, *Oscillibacter*, and *Anaerostipes*) and a lower abundance of pro-inflammatory bacteria (e.g., *Tyzzerella* and *Alistipes*) in the OB-CLA group compared with the OB group. Our correlation analysis suggested that the change in gut microbiota modified the hepatic FA composition and improved liver inflammation and intestinal permeability in OB mice.

Preclinical and human studies have suggested the beneficial effects of CLAs against cancer, obesity, and atherosclerosis (2). However, CLA has about 28 different isomers, and the two major isomers (*cis*-9, *trans*-11, and *trans*-10, *cis*-12) exhibited different fat-reducing properties. Thus, the purity and isomer ratio of CLA are key factors in determining its biological functions. Commercially available CLA products are commonly synthesized from linoleic acid (LA) by alkaline isomerization (29). In this study, urea-inclusion crystallization with a 2-step process was used to yield CLA with a purity of 94.68%. Moreover, the purified CLA contained more *trans*-10 and *cis*-12 CLA isomers. CLA treatment resulted in an effective anti-obesity effect in OB mice, and this function was mainly exhibited by *trans*-10, *cis*-12 CLA (2, 34). Thus these results further confirmed that the type and ratio of CLA isomers were important to its biological function.

As with most studies on CLA, body weight, fat tissue mass, and serum lipid levels were significantly downregulated by highly purified CLA in WT and OB mice (2). However, the controversial topics were that CLA treatment induced hepatic steatosis in the WT mice (3, 4) and reversed the obesity-induced hepatic lipid accumulation in the OB mice (8), which indicated that the effect of CLA on the liver was related to body conditions. Although some studies have suggested that decreased leptin and subsequent insulin resistance might be the reasons for hepatic steatosis induced by CLA (5–7), leptin deficiency in the ob/ob mice did not aggravate lipid accumulation in the OB-CLA group, which was similar to a previous study (8).

In this study, liver lipid analysis showed that there were more saturated FAs in the WT-CLA group and more unsaturated FAs in the OB-CLA group. Martha et al. found that in mice with normal food, CLA increased 14:0, 16:0, and 18:0 FA composition of the liver and reduced arachidonate (20:4;5,8,11,14) (35). In obese rats with a high-fat diet, CLA supplementation induced an increase in the polyunsaturated FAs in the liver lipids (36). The data suggest that CLA affected hepatic lipid metabolism in these mice. Liang et al. reported that liver inflammation causes dysfunction of hepatic lipid metabolism in rodents (37). Our results also showed that CLA treatment modified liver inflammation by increasing the mRNA expression of TNF-α and IL-6 in the WT mice with lipid accumulation, while reducing TNF-α, IFN-γ, and IL-1β in the OB mice. The liver has a dual blood supply from the hepatic arteries and portal vein. Since serum lipid levels were decreased by CLA in WT mice, the main source of lipids accumulated in the liver might has originated from the portal vein. It is well known that the portal vein transports substances absorbed from the stomach and intestine; thus, we further tested intestinal permeability and gut microbiota.

Previous animal experiments suggest that higher intestinal permeability and subsequent intraportal LPS infusion are major mechanisms of fatty liver disease (38). Antje et al. also confirmed that the extent of liver steatosis in humans is related to intestinal permeability (39). This permeability of the intestine depends on tight junctions composed of specialized proteins, such as occludin, ZO-1, claudins, and junctional adhesion molecules (40). In this study, CLA decreased the mRNA and protein expressions of occludin of the proximal colon in WT mice, which may weaken intercellular adhesion and increase permeability. In the OB mice, CLA increased the lower mRNA and protein expression of occludin and ZO-1 to recover intestinal permeability. Combined with the results of liver inflammation, we speculate that higher intestinal permeability in the WT-CLA group was accompanied by an increased liver inflammation response, and low-grade liver inflammation was observed in the OB-CLA group with recovered intestinal permeability. Therefore, the different intestinal permeability between the CLA-treated and untreated groups might be an important factor influencing CLA-induced hepatic steatosis.

The gut microbiota have been found to play a major role in the weight loss induced by CLA. Den Hartigh et al. reported that weight loss and divergent metabolic effects of mice fed t10c12-CLA or food restriction were associated with compositional differences in the gut microbiota, including an abundance of *Allobaculum*, *Butyrivibrio*, and *Bifidobacteria* with t10c12-CLA and enrichment of *Bacteroides* by FR (41). In this study, CLA treatment significantly changed the abundance and composition of gut microbiota. At the bacterial genus level, the WT-CLA group had higher proportions of *Lactobacillus*, *Muribaculum*, and *Faecalibaculum* than the WT group. *Lactobacillus* is a type of SLCFA-producing bacteria (42); thus, the higher composition of saturated FAs in the livers of the WT-CLA group might have originated from the intestine. *Muribaculum* and *Faecalibaculum* have been reported to be pro-inflammatory and anti-inflammatory genera, respectively (43, 44), and they might be related to the elevation of mRNA expression of TNF-α and IL-6 in the WT-CLA group. *Tyzzerella* and *Alistipes*, the typical pro-inflammatory bacteria (45), were abundant in the OB group, which improved the opportunity to induce the hyperinflammatory state of the liver via portal vein. In the OB-CLA group, CLA not only decreased the abundance of these pro-inflammatory bacteria but also significantly increased the abundance of *Lachnoclostridium*, *Roseburia*, *Dubosiella*, *Oscillibacter*, and *Anaerostipes*, which are believed to be beneficial for health for their anti-inflammatory and anti-oxidative stress activities, short-chain FA production, polyunsaturated FA transport, and other properties (46–48). The altered abundances of bacterial genera in the different groups were statistically related to intestinal permeability, liver inflammation, and hepatic lipid levels, suggesting that the hepatic steatosis induced by CLA is complex and multifactorial.

In conclusion, the findings from this study suggest a novel mechanism of hepatic lipid accumulation induced by CLA by influencing intestinal barrier function and gut microbial distribution (Fig. 6b). Therefore, understanding and applying highly purified CLA may be beneficial in promoting its anti-obesity and fatty liver-preventing effects.
